# Supplementary material for: The Identification of a Novel Nucleomodulin MbovP467 of Mycoplasmopsis bovis and Its Potential Contribution in Pathogenesis
Source: Cells. 2024 Mar 29;13(7):604. doi: 10.3390/cells13070604 (PMC11011252; doi:10.3390/cells13070604)
Supplement: Supplementary file 1 [file cells-13-00604-s001.zip › Supllementary Figures S1,S2,S3.pdf]

Mbov_0467

Prediction: Signal peptide (Sec/SPI)

Cleavage site between pos. 27 and 28: LLI-KK. Probability: 0.0607

Protein type	Signal peptide (Sec/SPI)	TAT signal peptide (Tat/SPI)	Lipoprotein signal peptide (Sec/SPII)	Other
Likelihood	0.5726	0.006	0.0998	0.3216

Download: PNG / EPS / Tabular

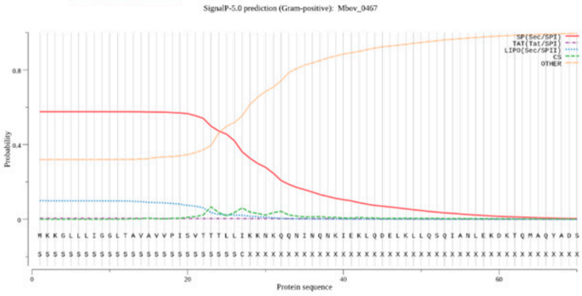

Figure S1: We predicted that the MbovP467 protein contained Signal peptide (Sec/SPI) but not Lipoprotein signal peptide (Sec/SPII) using the signal 5.0 online website.

cNLS Mapper Result

Predicted NLSs in query sequence

```
MKKGLLLIGGLTAVAVVPISVTTTLLIKKNKQQNINQNKIEKLQDELKLL 50
QSQIANLEKDKTQMAQYADSLIYSFDIENYQLESLEAMLKLQAKFHKLNS 100
YVDELKNQISIKKQNVTELEKEIKRLRNELSHDRNAIRFEVQRLVTDEWA 150
NMKDEILQSHKVSDIVKHLNKRIKFTKLPYQIKTDSDKTIKSLKDATKNL 200
ILSFDGLDFELTLELKDVSFHLDSIEHKYEDSQETICKIIGYYKDGSGKI 250
AVKPFAKSTKKVPTRLPWFIESLKAAFKDNKSSNIENLNEWNTSNVTDMS 300
MMFEASQINQPIRFDTRNVITMYSMFYEAKHFNSPLNFDTRNVQNMKAMF 350
YDALEFDQELKFNTKNVTDMSLMFSGASKFNKPLNFDTKNVKKMNSMFWG 400
TNEFNQPINFNTQNVEDIEQMFSHAKAFNQILNFDTRNVTNMRGLLELAE 450
NFNSNLNFSDTQNVTTMEMMFNGAINFNKPINFNTKKVTNMKFMFNNAYK 500
FNSPIKFDTNNVTNMYGMFYGALEFNQPLNFNTSNVENMGNMFYNAKKFN 550
SELKFSNTRNVKDMSGMFCYAEAFNQPLDFDTRNLENIKWMFYDAKNFNS 600
KLNFIDTSKIKNMQGAFQKASKFNQDISNWNIQAVTDFSDMFEGANAFKQ 650
DLSKWKSNPNWK                                       662
```

Predicted monopartite NLS

Pos.	Sequence	Score
119	LEKEIKRLRNE	3.5
166	VKHLNKRIKFTKLPY	4.5

Predicted bipartite NLS

Pos.	Sequence	Score
2	KKGLLLIGGLTAVAVVPISVTTTLLIKK	2
1	MKKGLLLIGGLTAVAVVPISVTTTLLIK	2.4
27	IKKNKQQNINQNKIEKLQDELKLLQSQIA	2.4
27	IKKNKQQNINQNKIEKLQDELKLLQSQIANLE	2.2
35	INQNKIEKLQDELKLLQSQIANLEKDKTQMA	2
86	EAMLKLQAKFHKLNSYVDELKNQISIKKQNVT	4.7
109	ISIKKQNVTELEKEIKRLRNELSHDRNAIRFE	2.3
148	EWANMKDEILQSHKVSDIVKHLNKRIKFTKLP	3.7
164	DIVKHLNKRIKFTKLPYQIKTDSDKTIKSLK	2.2
167	KHLNKRIKFTKLPYQIKTDSDKTIKSLKD	5.4
167	KHLNKRIKFTKLPYQIKTDSDKTIKSLKDATK	2.6
170	NKRIKFTKLPYQIKTDSDKTIKSLKDAT	2.4
190	IKSLKDATKNLILSFDGLDFELTLELKDVSF	3.3
214	ELKDVSFHLDSIEHKYEDSQETICKIIGYY	4.3
234	ETICKIIGYYKDGSGKIAVKPFAKSTKKVPT	3.6
249	KIAVKPFAKSTKKVPTRLPWFIESLKAAFKD	2.5
257	KSTKKVPTRLPWFIESLKAAFKDNKSSNIEN	2.4
274	KAAFKDNKSSNIENLNEWNTSNVTDMSMMFE	2.1
559	RNVKDMSGMFCYAEAFNQPLDFDTRNLENIK	2
583	RNLENIKWMFYDAKNFNSKLNFIDTSKIKNMQG	3.9

Figure S2 A prediction NLS sequence was identified using cNLS Mapper (cut-off score≥5)

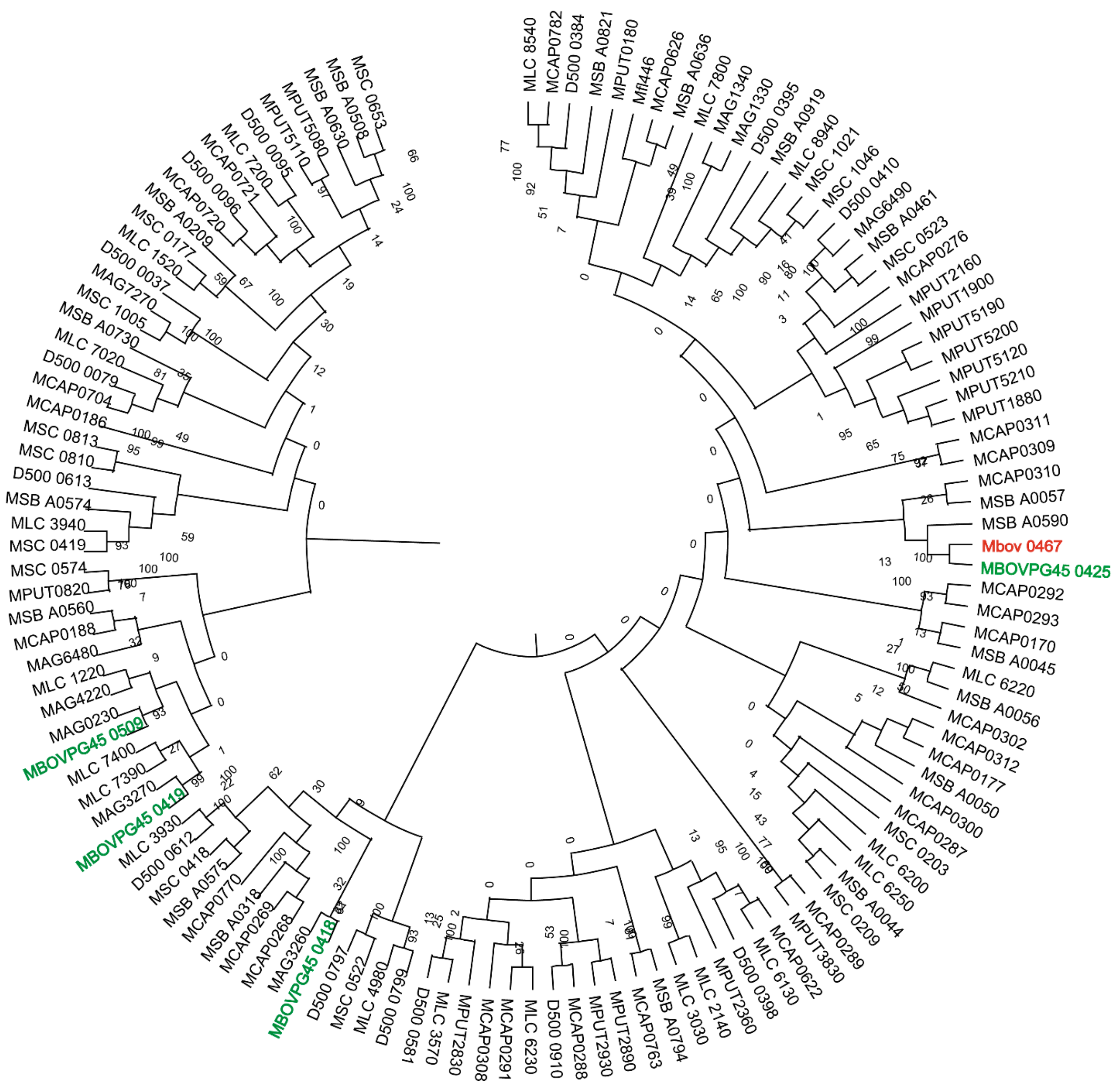

Figure S3: The MolliGen, 3.0 web server was used to identify homologous proteins of MbovP467 (red color) has a homolog in *Mycoplasmopsis bovis* PG45, including MBOVPG45 0425MBOVPG45 0418, MBOVPG45 0419 and MBOVPG45 0509 (green color). MbovP467 is not only present in HB0801 but also in *Mycoplasmopsis bovis* PG45 standard strain and many other mycoplasmas species, including *Mycoplasmopsis feriruminatoris*, *Mycoplasmopsis leachii*, *Mycoplasmopsis mycoides* subsp. *Capri*.
