# Supplementary material for: The Identification of a Novel Nucleomodulin MbovP467 of Mycoplasmopsis bovis and Its Potential Contribution in Pathogenesis
Source: Cells. 2024 Mar 29;13(7):604. doi: 10.3390/cells13070604 (PMC11011252; doi:10.3390/cells13070604)
Supplement: Supplementary file 1 [file cells-13-00604-s001.zip › Supplementary table legend.pdf]

Table S1: All DEGs during treatment

Table S2: DEGs upregulated genes

Table S3: DEGs Downregulated
